# Spin Seebeck effect of correlated magnetic molecules

**DOI:** 10.1038/s41598-021-88373-7

**Published:** 2021-04-28

**Authors:** Anand Manaparambil, Ireneusz Weymann

**Affiliations:** Institute of Spintronics and Quantum Information, Faculty of Physics, Adam Mickiewicz University in Poznań, ul. Uniwersytetu Poznańskiego 2, 61-614 Poznań, Poland

**Keywords:** Molecular electronics, Spintronics

## Abstract

In this paper we investigate the spin-resolved thermoelectric properties of strongly correlated molecular junctions in the linear response regime. The magnetic molecule is modeled by a single orbital level to which the molecular core spin is attached by an exchange interaction. Using the numerical renormalization group method we analyze the behavior of the (spin) Seebeck effect, heat conductance and figure of merit for different model parameters of the molecule. We show that the thermopower strongly depends on the strength and type of the exchange interaction as well as the molecule’s magnetic anisotropy. When the molecule is coupled to ferromagnetic leads, the thermoelectric properties reveal an interplay between the spin-resolved tunneling processes and intrinsic magnetic properties of the molecule. Moreover, in the case of finite spin accumulation in the leads, the system exhibits the spin Seebeck effect. We demonstrate that a considerable spin Seebeck effect can develop when the molecule exhibits an easy-plane magnetic anisotropy, while the sign of the spin thermopower depends on the type and magnitude of the molecule’s exchange interaction.

## Introduction

Thermoelectric properties of nanoscale systems have recently attracted a considerable attention^1–5^. This is due to the fact that such systems are expected to offer much better thermoelectric efficiency as compared to their bulk counterparts^6–8^. Moreover, it turns out that studying the behavior of thermoelectric coefficients can provide additional information about various correlations and quantum interference present in the system^9–16^. One prominent example resulting from electronic correlations is the Kondo effect^17,18^ observed in quantum dots and molecules^19,20^, for which the sign changes of the thermopower have been proposed as additional signatures and measures of the strength of Kondo correlations^21–24^. In fact, thermoelectric properties of Kondo-correlated nanoscale junctions have been recently explored experimentally^10,25,26^.

Interestingly, the thermoelectric phenomena of nanostructures have also been explored in the case of systems involving magnetic components. In fact, with the discovery of the spin Seebeck effect^27^, a new field of interest, namely spin caloritronics, have started blossoming^28–32^. It transpires that the interplay of charge, heat and spin gives rise to a rich behavior of the thermoelectric coefficients that are now spin-dependent^33–35^. In correlated magnetic nanostructures, such as e.g. quantum dots coupled to ferromagnetic leads, the spin thermopower was shown to provide further information about an exchange field and its interactions with electronic correlations driving the Kondo effect^36,37^. Moreover, the spin Seebeck effect has also been studied in the case of quantum dots subject to external magnetic field^38–40^. An interesting situation occurs when the junction comprises a molecule of large spin, since the spin Seebeck effect is then additionally conditioned by intrinsic parameters of the molecule, such as an exchange interaction, magnetic anisotropy or the magnitude of the molecule’s spin^41,42^. In fact, the spin-dependent thermoelectric properties of large-spin molecular junctions have already been studied in the case of weak coupling to the contacts^43–46^, whereas the system’s behavior in the strongly correlated case remains to a large extent unexplored. This comprises the goal of this paper, which is to further extend the understanding of thermoelectricity in strongly correlated magnetic molecular systems.

We therefore undertake the studies of the Seebeck and spin Seebeck effects for a large-spin molecule, such as a single molecular magnet^47–49^, embedded in a tunnel junction with either nonmagnetic or ferromagnetic contacts. The focus is on the linear response regime with respect to the applied potential and temperature gradients, which justifies the usage of the numerical renormalization group (NRG) method^50,51^ for the calculations. This method allows for obtaining very accurate results for the electrical and heat conductances as well as the Seebeck effect and the corresponding figure of merit in the full parameter space of the model. The molecule is assumed to possess an orbital level, through which transport takes place, which is exchange coupled to the spin of the molecule’s internal core^52–54^. First of all, we show that the Seebeck coefficient strongly depends on the type and strength of the exchange interaction. In particular, for antiferromagnetic exchange, we find an additional sign change of the thermopower as a function of temperature. Moreover, in the case of magnetic contacts, the interplay of Kondo screening with exchange field determines the thermoelectric response of the system. We consider two specific cases regarding the spin relaxation time in the leads^35,36^. In the case of slow spin relaxation, a spin bias can be generated in the system, which gives rise to the spin Seebeck effect. On the other hand, for fast spin relaxation, the spin Seebeck effect does not develop, however, the thermoelectric coefficients still exhibit interesting spin-resolved properties due to spin dependence of tunneling processes. We believe that our study, by providing a comprehensive analysis of (spin) thermopower in the case of large-spin molecules, adds a new insight into the interplay of heat, charge and spin in magnetic molecules, contributing thus to further development of molecular spin caloritronics.

## Results

**Figure 1:**
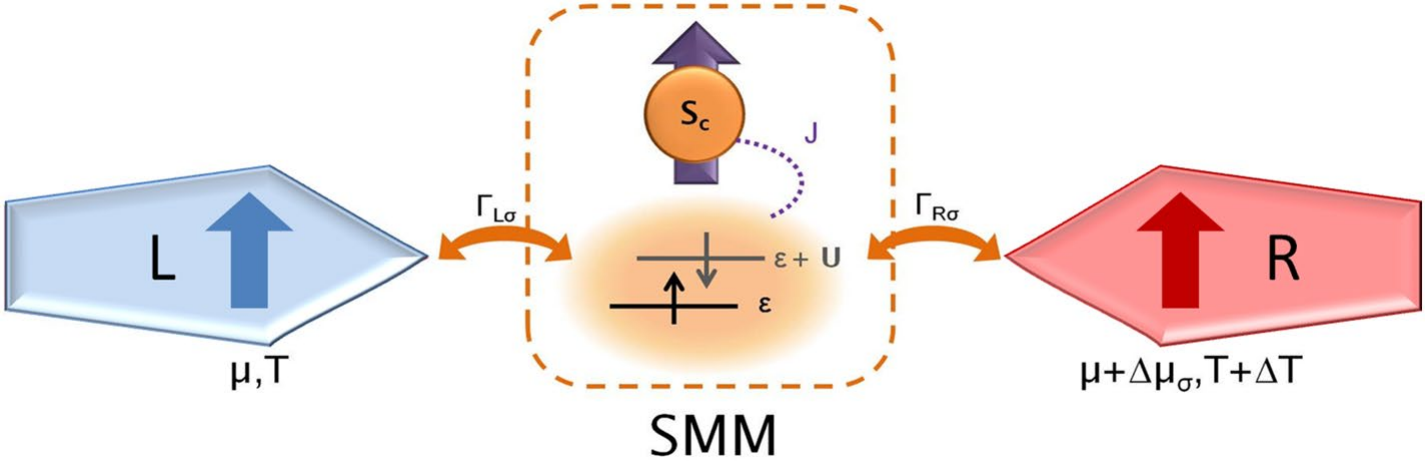
Schematic of the considered molecular junction. It consists of a magnetic molecule of spin $S=S_c+s$ tunnel-coupled to external leads, with spin-dependent coupling strengths, $\Gamma _{L\sigma }$ and $\Gamma _{R\sigma }$, for the left and right lead. $S_c$ is the spin of the molecule’s magnetic core, while *s* denotes the spin of electrons occupying the orbital level. The molecule is assumed to effectively possess one orbital level, through which transport takes place, that is exchange-coupled (with coupling strength *J*) to the molecule’s core spin $S_c$. The orbital level is characterized by on-site energy $\varepsilon$ and the Coulomb correlations *U*. There is a voltage ($\Delta \mu _\sigma$) and temperature ($\Delta T$) gradient applied to the system. In the case of magnetic contacts, the voltage gradient may be spin-dependent. In considerations we assume $S_c = 1$, while $s=1/2$ ($s=0$) when the orbital level occupancy is odd (even).

The schematic of the studied system is presented in Fig. 1. A high-spin magnetic molecule, of spin $S=S_c+s$, is coupled to external magnetic leads, whose magnetizations point in the same direction. The molecule’s easy axis is assumed to coincide with the direction of leads’ magnetizations. It is further assumed that a single molecular energy level is active in transport and this orbital level is coupled through an exchange interaction *J* to the core spin $S_c$ of the molecule. Thus, the Hamiltonian of the molecule reads as^52–54^,

1 $$\begin{aligned} \hat{H}_{\text {mol}} = \varepsilon \sum _\sigma \hat{n}_\sigma + U\hat{n}_\uparrow \hat{n}_\downarrow - J\hat{\mathbf {S}}_\mathbf {c}\cdot \hat{\mathbf {s}} - D \hat{S}_z^2, \end{aligned}$$

where $\varepsilon$ and *U* denote the energy of the molecule’s orbital level and Coulomb correlation energy between two electrons of opposite spin occupying that level. $\hat{n}_\sigma \equiv \hat{d}_\sigma ^\dag \hat{d}_\sigma$ is the occupation number operator for an electron of spin $\sigma$ and $\hat{d}_\sigma ^\dag$ ($\hat{d}_\sigma$) is the corresponding creation (annihilation) operator. The spin operator for an electron occupying the orbital level is denoted by $\hat{\mathbf {s}}\equiv (1/2)\sum _{\sigma \sigma '}\hat{d}_\sigma ^\dag \mathbf {\sigma }_{\sigma \sigma '}\hat{d}_{\sigma '}$, where $\mathbf {\sigma }\equiv (\sigma ^x,\sigma ^y,\sigma ^z)$ denotes the vector of the Pauli matrices, and $\hat{\mathbf {S}}_\mathbf {c}$ is the operator for the core spin of the molecule. The two spins are coupled by the exchange interaction *J*, which can be either of ferromagnetic ($J>0$) or antiferromagnetic ($J<0$) type, depending on the sign of *J*. The molecule can be subject to magnetic anisotropy denoted by *D* and $\hat{S}_z$ is the *z*th component of the molecule spin operator $\hat{\mathbf {S}}=\hat{\mathbf {S}}_\mathbf {c}+\hat{\mathbf {s}}$. In calculations, we assume $S_c=1$, while the spin of electrons on the orbital level is given by $s=1/2$ or $s=0$, depending on its occupancy. Consequently, the total molecule’s spin is $S=3/2$ for singly occupied orbital level or $S=1$ in the case when the occupation is even.

The tunneling processes between the molecule and the leads are described by the following Hamiltonian

2 $$\begin{aligned} \hat{H}_{\text {tun}} = \sum _{qk\sigma } v_{qk\sigma } (\hat{c}_{qk\sigma }^{\dagger } \hat{d}_{\sigma } + \hat{d}_\sigma ^\dag \hat{c}_{qk\sigma }), \end{aligned}$$

where $q=\mathrm{L}$ for the left and $q=\mathrm{R}$ for the right electrode, the operator $\hat{c}_{qk\sigma }^{\dagger }$ ($\hat{c}_{qk\sigma }$) creates (annihilates) an electron with energy $\varepsilon _{qk\sigma }$, momentum *k* and spin $\sigma$ in the *q*-th lead, and $v_{qk\sigma }$ denotes the corresponding tunnel matrix elements. The leads are described within the non-interacting quasi-particle approximation by

3 $$\begin{aligned} \hat{H}_{\text {leads}}=\sum _{qk\sigma } \varepsilon _{qk\sigma } \hat{c}_{qk\sigma }^{\dagger } \hat{c}_{qk\sigma }. \end{aligned}$$

Having defined the three parts of the Hamiltonian, the total Hamiltonian is given by, $\hat{H} = \hat{H}_{\text {mol}}+ \hat{H}_{\text {tun}}+\hat{H}_{\text {leads}}$.

The tunnel coupling between the molecule and the leads gives rise to the broadening of the orbital level, which can be described by, $\Gamma _{q\sigma } = \pi \rho _{q\sigma } v_{q\sigma }^2$, where $\rho _{q\sigma }$ is the spin-dependent density of states at the Fermi level in the lead *q* and we assumed momentum-independent tunnel matrix elements $v_{qk\sigma }\equiv v_{q\sigma }$. We then define the full broadening function, which for spin $\sigma$ can be written as, $\Gamma _\sigma = (1+\eta p) \Gamma$, where $\Gamma = \Gamma _L + \Gamma _R$ and $\Gamma _{q} = \Gamma _{q\uparrow }+ \Gamma _{q\downarrow }$, whereas *p* is the effective spin polarization of the left and right ferromagnetic lead, $p = (p_L + p_R)/2$, and $\eta =1$ ($\eta =-1$) for spin-up (spin-down) electrons.

### Thermopower in the case of nonmagnetic leads

We focus on the thermoelectric transport properties of the considered molecular junction in the linear response regime. The interesting thermoelectric phenomena happening across the magnetic molecule can be quantified using the transport coefficients, such as the electrical conductance *G*, the thermopower (Seebeck coefficient) *S*, as well as the thermal conductance $\kappa$ and the thermoelectric figure of merit *ZT*. In the linear response regime, these quantities can be expressed in terms of Onsager integrals $L_{n\sigma }$^55^

4 $$\begin{aligned} L_{n\sigma } = -\frac{1}{h}\int d\omega (\omega -\mu )^{n} \frac{\partial f}{\partial \omega } \mathscr {T}_{\sigma }(\omega ), \end{aligned}$$

where $\mathscr {T}_\sigma (\omega )$ is the energy-dependent transmission coefficient for the spin channel $\sigma$, *f* is the Fermi–Dirac distribution function and $\mu$ denotes the electrochemical potential. The electrical conductance *G* and the electronic contribution to the thermal conductance $\kappa$ can be then found from

5 $$\begin{aligned} G=e^2 L_{0} \;\;\;\;\; \mathrm{and} \;\;\;\;\; \kappa =\frac{1}{T} \left( L_{2} - \frac{L_{1}^2}{L_{0}} \right) , \end{aligned}$$

where *e* is the electron charge, *T* denotes the temperature and $L_n=\sum _\sigma L_{n\sigma }$. On the other hand, the thermopower *S* is defined as $S=-[\Delta V/\Delta T]_{J=0}$, on the condition of vanishing of the charge current *J*. Hence, *S* can be expressed in the form

6 $$\begin{aligned} S=-\frac{1}{|e| T}\frac{L_{1}}{ L_{0}} . \end{aligned}$$

Having defined *G*, *S* and $\kappa$, one can obtain the thermoelectric figure of merit $ZT\equiv GS^2T/\kappa$.

**Figure 2:**
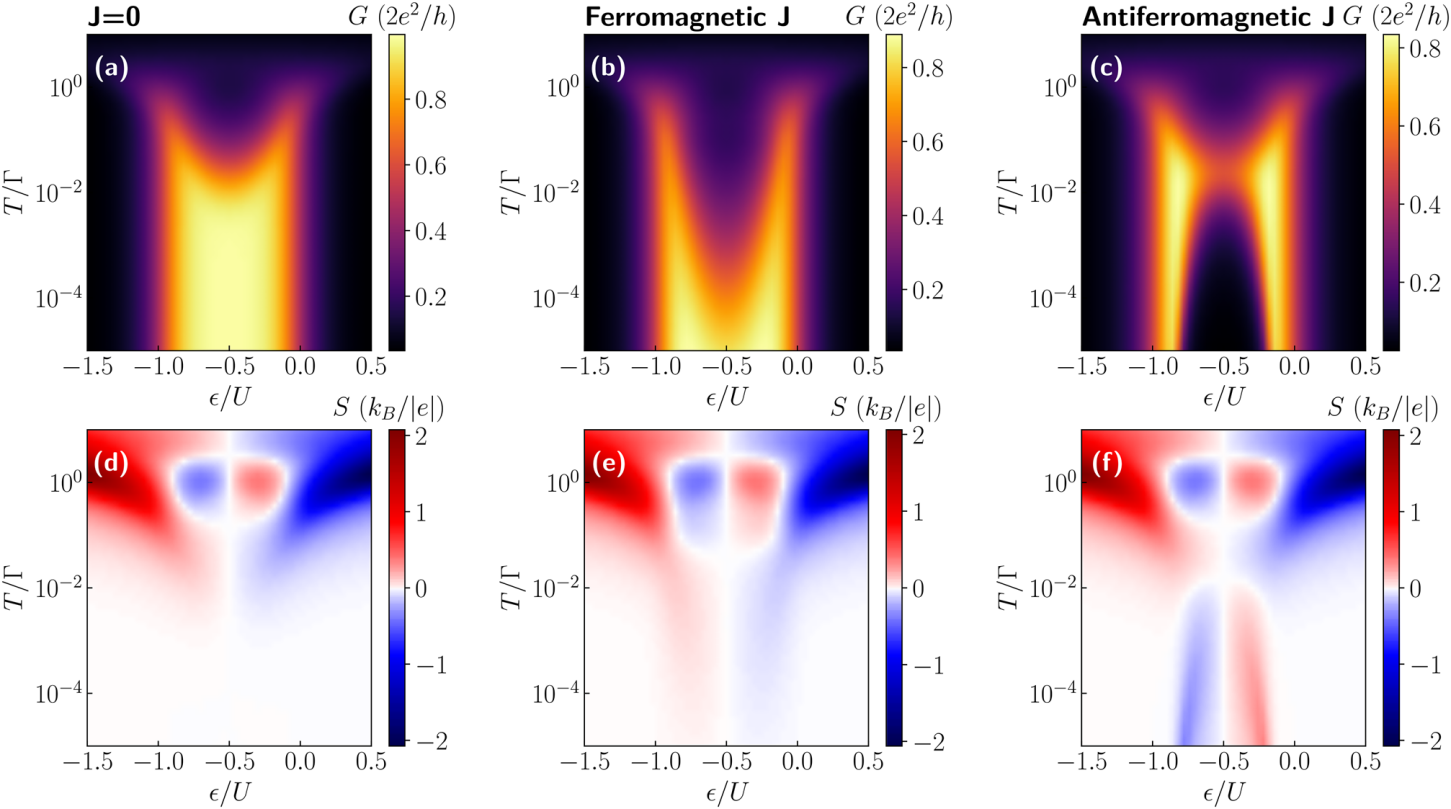
Conductance and thermopower of molecule in nonmagnetic junction. The linear conductance *G* and thermopower *S* as a function of the position of molecule’s level $\varepsilon$ and temperature *T* in the case of (**a**,**d**) $J=0$, (**b**,**e**) ferromagnetic ($J=10T_{\mathrm{K}}$) and (**c**,**f**) antiferromagnetic ($J=-T_{\mathrm{K}}$) exchange interaction, where $T_{\mathrm{K}}= 0.0022$ is the Kondo temperature in the case of $\varepsilon =-U/2$, $J=0$ and $p=0$. The other parameters are: $\Gamma = 0.05$, $U = 0.5$, $D=0$, in units of band halfwidth, and $p=0$. The spin of the molecule is equal to $S=3/2$.

Let us first consider the case of nonmagnetic molecular junction. The linear conductance and the Seebeck coefficient as a function of the molecule’s orbital level energy $\varepsilon$ and temperature *T* are shown in Fig. 2. The first column shows the results for $J=0$, while the second (third) column corresponds to the case of the ferromagnetic (antiferromagnetic) exchange interaction *J*. The case of $J=0$ is shown just for reference and allows us to clearly reveal the effects stemming from the presence of large-spin molecule. To begin with, we consider the behavior of the linear conductance. The largest changes with lowering the temperature are visible when the orbital level is singly occupied, i.e. for $-U \lesssim \varepsilon \lesssim 0$. In this case, the Kondo effect can develop at sufficiently low temperatures, such that $T\lesssim T_K$, where $T_K$ is the Kondo temperature^17^. Once $T\ll T_K$, the conductance reveals a plateau as a function of $\varepsilon$ of height $G = 2e^2/h$ in the case of $J=0$^18,20^. However, for magnetic molecules described by the Hamiltonian (1), the low-temperature behavior strongly depends on the type of exchange interaction *J*^56^. For ferromagnetic exchange, the Kondo effect always develops with lowering *T*, however, $T_K$ becomes reduced compared to the case of $J=0$. On the other hand, in the antiferromagnetic-*J* case, once $|J|\gtrsim T_K$, the spin on the orbital level strongly binds with the magnetic core spin, which results in the suppression of the conductance through the system. In this case, in the singly occupied orbital regime, one only observes a small enhancement (a local maximum) of *G* with decreasing *T* followed by its strong suppression^56^.

The different scenarios discussed above give rise to a unique behavior of the Seebeck coefficient, which is presented in the bottom row of Fig. 2. The first observation is that, as expected^9,12^, the thermopower changes sign with respect to the particle-hole symmetry point, $\varepsilon = -U/2$. Moreover, one can see that the behavior of *S* for $T\gtrsim \Gamma$ is hardly affected by the type of exchange interaction *J*. This is because in our considerations $|J|<\Gamma$ and the effects of finite *J* can be visible only when $T\lesssim |J|$. Let us anyway summarize the main features of the high-temperature behavior. One observes two pronounced maxima for $T\approx \Gamma$ in the case when the orbital level is either empty or doubly occupied. On the other hand, when moving to the Coulomb blockade regime where the orbital level is singly occupied, the thermopower changes sign and two local extrema develop antisymmetrically around $\varepsilon =-U/2$ for $T\approx \Gamma$^21,36^. With lowering the temperature, distinct features appear, resulting from the interplay of the correlations driving the Kondo effect and the molecule’s exchange interaction. First of all, one can note that in the case of $J=0$, the regions of large |*S*| extend from the empty and doubly occupied regimes for $T\approx \Gamma$ downwards to low temperatures in the single occupancy regime. For singly occupied orbital level, the thermopower exhibits then a sign change as a function of *T*, see Fig. 2d, which signals the relevance of the Kondo correlations^21^. A qualitatively similar behavior can be observed in the case of ferromagnetic exchange interaction *J*, see Fig. 2e, with the main difference associated with smaller temperatures at which the corresponding sign change occurs. This is associated with the fact that the sign change occurs at the onset of the Kondo correlations and, because the Kondo effect develops at much lower temperatures in the case of ferromagnetic *J* compared to the case of $J=0$ (see Fig. 2a,b), one observes that the sign change in *S* is also shifted to lower temperatures. However, this shift is not proportional to the corresponding shift visible in the behavior of *G*. It is because while much smaller temperatures are needed for the full development of the Kondo effect in the case of ferromagnetic *J*, the temperature associated with the onset of the Kondo correlations only weakly decreases with *J*. This is why the crossover for $J>0$ is only slightly shifted to lower temperatures compared to the case of $J=0$, cf. Fig. 2d,e. Interestingly, qualitatively new features compared to the case of $J\ge 0$ can be observed in the case of antiferromagnetic exchange interaction, where an additional sign change at low temperatures is present, see Fig. 2f.

**Figure 3:**
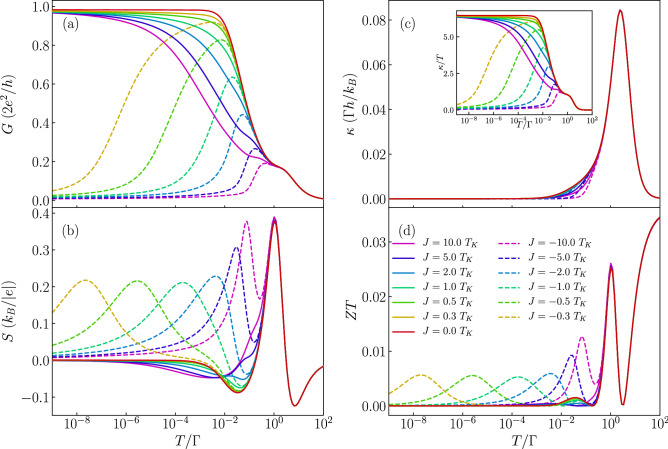
Dependence of thermoelectric coefficients on exchange interaction. (**a**) The conductance, (**b**) Seebeck coefficient, (**c**) heat conductance and (**d**) figure of merit as a function of temperature for selected values of exchange interaction *J* in the case of nonmagnetic contacts. The solid (dashed) lines correspond to the case of ferromagnetic (antiferromagnetic) exchange interaction. The inset in (**c**) presents the temperature dependence of $\kappa /T$. The parameters are the same as in Fig. 2 with $\varepsilon =-U/3$.

Further insight into the behavior of the thermoelectric properties can be obtained from the inspection of Fig. 3, which presents the temperature dependence of *G*, *S*, $\kappa$ and *ZT* for different values of the exchange interaction *J*, as indicated. This figure is generated for the case when the orbital level is detuned from the particle-hole symmetry point, such that a considerable thermopower can be observed. In addition, the orbital level is assumed to be singly occupied ($\varepsilon =-U/3$), such that the system is in the local moment regime and the Kondo correlations are relevant at sufficiently low temperatures. Let us start with the analysis of the linear conductance. In the case of ferromagnetic *J* (see the solid lines in the figure), the enhancement of *J* results in a decrease of the Kondo temperature. In this case the ground state is always two-fold degenerate with total spin given by $S = S_c+1/2$ and the Kondo effect develops irrespective of *J*, though $T_K$ becomes very cryogenic with increasing *J*^56^. Consequently, we observe mainly quantitative changes in Fig. 3a, while qualitative behavior is the same. On the other hand, the situation is completely different in the case of antiferromagnetic exchange interaction $J<0$, see the dashed lines in Fig. 3. Now, with increasing |*J*|, the ground state becomes $S = S_c-1/2$, since the spin on the orbital level binds anti-ferromagnetically with the molecule’s core spin. Because of that, the Kondo effect is quenched once the temperature becomes lower than the energy scale responsible for this antiferromagnetic state. As a result, the temperature dependence of conductance exhibits a nonmonotonic behavior^56^, see Fig. 3a. We note that such behavior is similar to the two-stage Kondo effect observed in side-attached double quantum dots where the hopping induces an antiferromagnetic interaction between the dots^57^.

The behavior of the conductance has a strong influence on the Seebeck coefficient. This is because the temperature dependence of conductance reflects the energy dependence of the transmission coefficient $\mathscr {T}(\omega )$ and, from the Sommerfeld expansion, the thermopower at low temperatures can be estimated from (note that temperature *T* is in units of energy)

7 $$\begin{aligned} S \approx - \frac{\pi ^2}{3} \frac{k_B}{e} \frac{T}{\mathscr {T}(\omega )} \left. \frac{\partial \mathscr {T}(\omega )}{\partial \omega } \right| _{\omega =0}, \end{aligned}$$

where $\mathscr {T}(\omega ) = \sum _\sigma \mathscr {T}_{\sigma }(\omega )$. One can thus see that the thermopower is related to the monotonicity of the variation of the spectral function with energy. This is why in the case of ferromagnetic exchange interaction we only observe qualitative changes in *S*, see the solid lines in Fig. 3b. The enhancement of *J* suppresses then the Kondo temperature, which is seen in the behavior of *S* as a shift of the local minimum towards smaller temperatures. However, a completely different scenario develops for antiferromagnetic *J*, where an additional sign change occurs and *S* exhibits an extra maximum for temperatures corresponding to the energy scale at which *G*(*T*) starts decreasing with lowering *T*, see the dashed curves in Fig. 3b.

Despite a spectacular impact of exchange interaction on the thermopower, its effect on the thermal conductance is less pronounced, see Fig. 3c. This is because $\kappa$ is considerable only at energy scales corresponding to the coupling strength and Coulomb correlations and thus, as long as $|J|\ll \Gamma$, $\kappa$ hardly depends on the type and magnitude of exchange interaction. On the other hand, the figure of merit *ZT* displays new peaks in the case of antiferromagnetic *J*, see Fig. 3d, which are associated with the above-discussed maxima emerging in the temperature dependence of *S*. We also note that the influence of *J* on $\kappa$ is more visible when one plots $\kappa /T$, see the inset in Fig. 3c. It is nicely visible that the qualitative behavior of $\kappa /T$ resembles that of the linear conductance, which is a direct consequence of the Wiedemann–Franz law. A similar behavior has been recently observed for T-shaped double quantum dots^23^.

#### Effect of magnetic anisotropy

**Figure 4:**
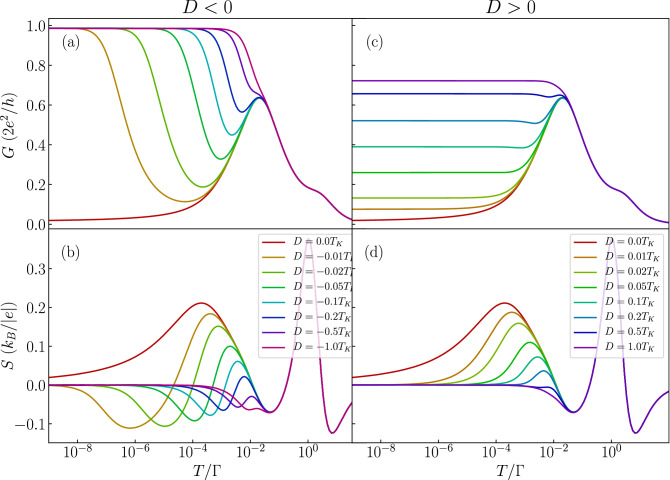
The effect of magnetic anisotropy in the case of molecule with nonmagnetic contacts. The temperature dependence of (**a**,**c**) the conductance and (**b**,**d**) the Seebeck coefficient in the case of antiferromagnetic exchange interaction $J=-T_K$ for selected values of magnetic anisotropy *D*. The left (right) column presents the case of easy-plane (easy-axis) type of magnetic anisotropy. The parameters are the same as in Fig. 2 with $\varepsilon =-U/3$.

We now focus on elucidating the role of magnetic anisotropy on the thermoelectric properties of the considered molecular junction. We consider both an easy-axis ($D>0$) and easy-plane ($D<0$) types of magnetic anisotropy. First we note that in the case of ferromagnetic exchange interaction between the orbital level and molecule’s core spin the magnetic anisotropy has a very moderate influence on the thermoelectric properties. It does not lead to new qualitative behavior as long as |*D*| is smaller than the corresponding Kondo temperature, therefore in the following we just analyze the case of antiferromagnetic exchange coupling *J*. The temperature dependence of the conductance and the Seebeck coefficient for this situation is shown in Fig. 4, where the left (right) column corresponds to the easy-plane (easy-axis) magnetic anisotropy case. This figure was generated for the same orbital level position as in Fig. 3, such that the orbital level is detuned from the particle-hole symmetry point, while the system stays in the local moment regime. In the absence of anisotropy the conductance displays a nonmonotonic dependence, characteristic of the antiferromagnetic exchange coupling. When an easy plane anisotropy arises in the system and the molecule possesses a half-integer spin, it results in a two-fold degenerate ground state of $S=1/2$, such that the Kondo effect can be restored. This is clearly seen in Fig. 4a, where one observes an upturn of the conductance with lowering *T*. This in turn has a considerable impact on the Seebeck coefficient, which exhibits an additional sign change, see Fig. 4b. On the other hand, in the case of easy plane anisotropy, the low-temperature conductance again becomes increased with *D*, as can be seen in Fig. 4c. This is however just associated with a decreased exchange interaction between the molecule’s core spin and the spin of the orbital level, and not with the reinstatement of the Kondo effect. Consequently, while the Seebeck coefficient strongly depends on *D*, no additional sign changes are present. In fact, the maximum in *S* for $D=0$ becomes suppressed with increasing *D* and smears out completely once $D\approx T_K$, see Fig. 4d.

### Thermopower in the case of ferromagnetic leads

**Figure 5:**
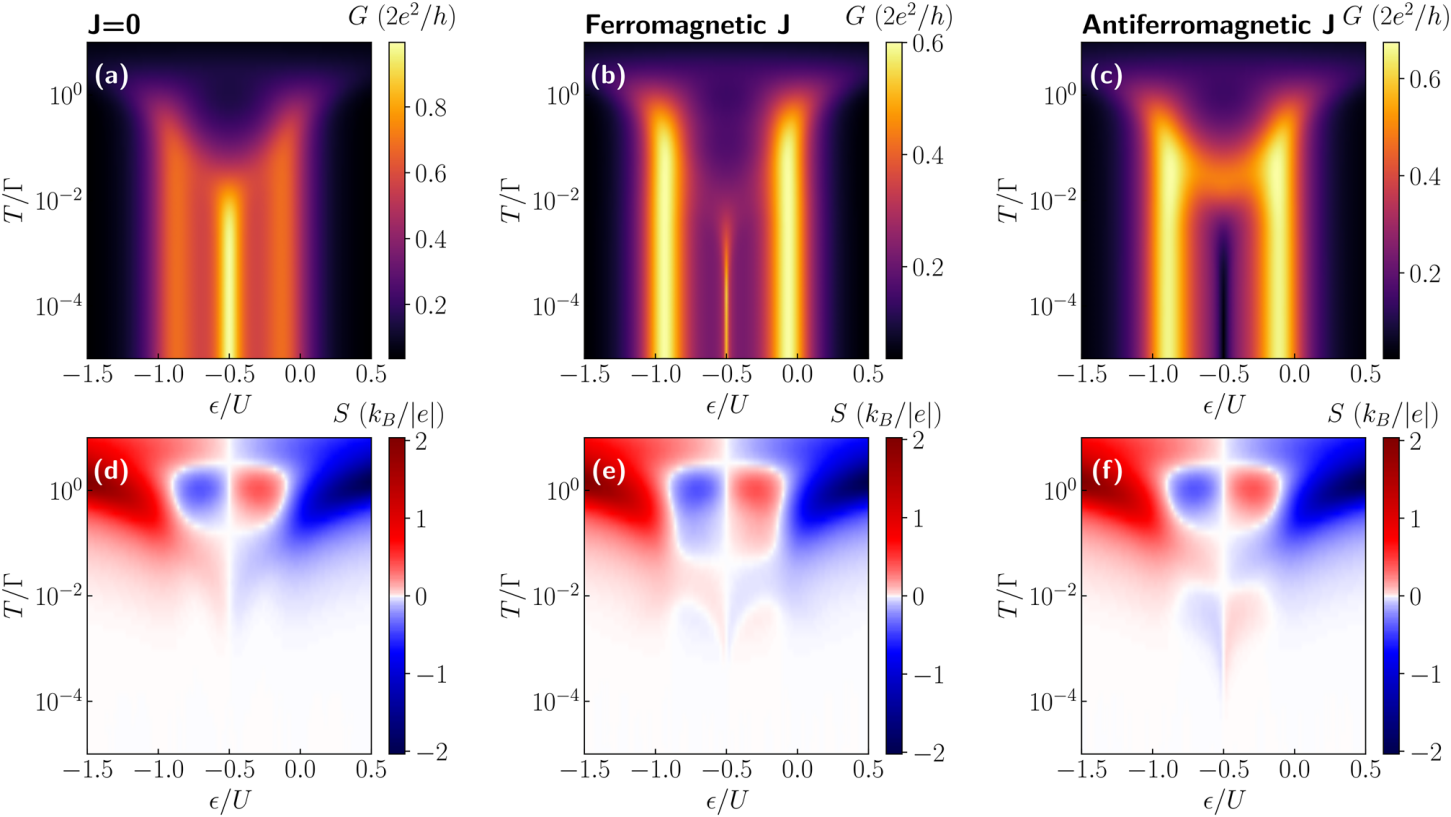
Conductance and thermopower of molecule in ferromagnetic junction. The linear conductance *G* and thermopower *S* as a function of the position of molecule’s level $\varepsilon$ and temperature *T* in the case of (**a**,**d**) $J=0$, (**b**,**e**) ferromagnetic ($J=10T_{\mathrm{K}}$) and (**c**,**f**) antiferromagnetic ($J=-T_{\mathrm{K}}$) exchange interaction. The other parameters are the same as in Fig. 2 with $p=20\%$.

We now turn to the discussion of thermoelectric properties in the case of ferromagnetic electrodes. For the ferromagnetic contacts, we assume a moderate spin polarization^58–60^, $p=20\%$. In this section we assume that the spin relaxation in the contacts is relatively fast, such that no spin accumulation develops and the induced potential gradient does not depend on spin, $\Delta \mu _\uparrow = \Delta \mu _\downarrow$. In this regime, although the spin Seebeck effect does not develop, the spin-dependence of tunneling processes greatly modifies the thermoelectric transport properties of the system as compared to the nonmagnetic case. We also note that in the absence of spin accumulation the formulas for spin-dependent thermoelectric coefficients are the same as in case of nonmagnetic leads^35,36^.

The linear conductance and the Seebeck coefficient as a function of $\varepsilon$ and *T* calculated for different values of *J* are displayed in Fig. 5. In the behavior of the conductance one can clearly observe the signatures of an effective exchange field that develops in the molecule coupled to ferromagnetic electrodes^54,61^. Such an exchange field, which within the perturbation theory at zero temperature and for $J=0$ can be described as^61^,

8 $$\begin{aligned} \Delta \varepsilon _{\mathrm{exch}}\approx \frac{2p\Gamma }{\pi }\text {log}\bigg |\frac{\varepsilon }{\varepsilon +U}\bigg |, \end{aligned}$$

results in a spin-splitting of the molecule’s orbital level, when it is detuned from the particle-hole symmetry point of the model $\varepsilon =-U/2$. In the case of considered molecule, this field depends in a nontrivial way on the properties of the molecule, such as *J*, *D* and $S_c$, however, it still vanishes whenever $\varepsilon =-U/2$^54,62^. If the exchange field splitting becomes larger than the Kondo temperature, it suppresses the Kondo resonance. As a consequence, the low-temperature conductance in the Coulomb blockade regime is generally decreased except for $\varepsilon =-U/2$, where a local maximum as a function of $\varepsilon$ is present, see Fig. 5a. A similar conductance suppression can be seen in the case of ferromagnetic exchange interaction *J* presented in Fig. 5b. However, now the suppression of *G* is larger as compared to the case of $J=0$, which is associated with a smaller Kondo temperature when $J>0$. On the other hand, when *J* is antiferromagnetic, mainly quantitative changes can be observed in the conductance behavior, cf. Figs. 2c and 5c. Namely, the region of suppression of low-temperature conductance is now smaller, which is an indication that the exchange field hinders the formation of an antiparallel spin state between the orbital level and the core spin. Because of that, the decrease of conductance due to that formation is correspondingly weakened. The signatures of the interplay of exchange field, Kondo correlations, and the molecule’s exchange interaction are also visible in the behavior of the thermopower, which is presented in the bottom row of Fig. 5. One can see that the main changes are visible in the low-temperature behavior, which reveals the energy scale associated with the exchange field. More specifically, in the case of ferromagnetic exchange interaction, an additional sign change in *S* occurs as compared to the case of nonmagnetic leads, whereas for antiferromagnetic *J* the Seebeck coefficient becomes generally reduced, cf. Figs. 2 and 5.

**Figure 6:**
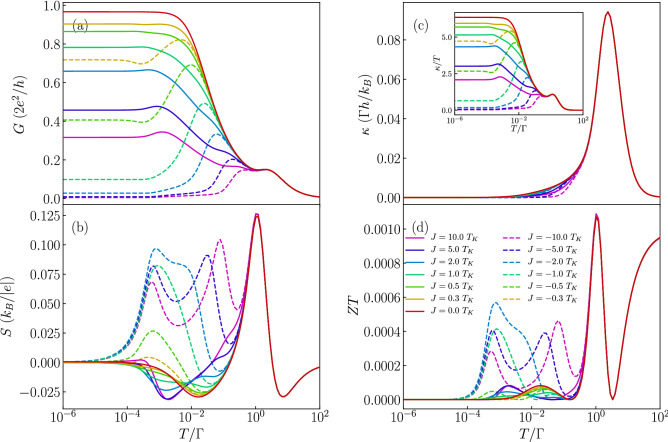
Dependence on exchange interaction in the case of ferromagnetic junction. (**a**) The conductance, (**b**) Seebeck coefficient, (**c**) heat conductance and (**d**) figure of merit as a function of temperature for selected values of exchange interaction *J* in the case of ferromagnetic leads. The solid (dashed) lines correspond to the case of ferromagnetic (antiferromagnetic) exchange interaction. The inset in (**c**) presents $\kappa /T$ as a function of temperature. The parameters are the same as in Fig. 5 with $\varepsilon =-0.48U$.

**Figure 7:**
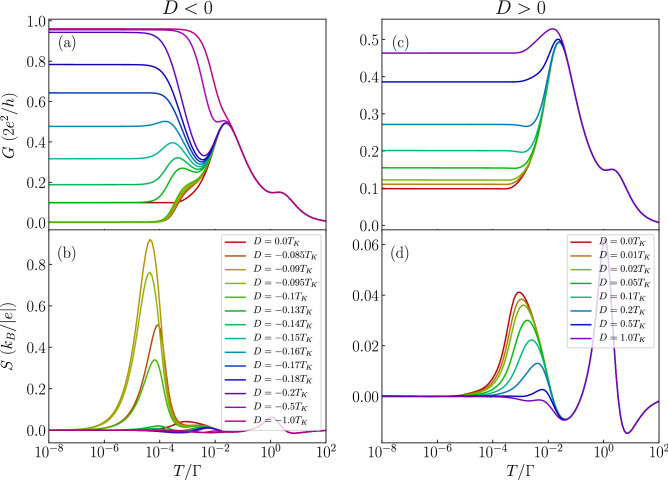
The effects of magnetic anisotropy in the case of ferromagnetic junction. The temperature dependence of (**a**,**c**) the conductance and (**b**,**d**) the Seebeck coefficient in the case of antiferromagnetic exchange interaction $J=-T_K$ for selected values of magnetic anisotropy *D*. The left (right) column presents the case of easy-plane (easy-axis) type of magnetic anisotropy. The parameters are the same as in Fig. 5 with $\varepsilon =-0.48U$.

To shed more light onto the thermoelectric behavior of the considered magnetic molecular junction, in Fig. 6 we present the temperature dependence of the conductance, Seebeck coefficient, heat conductance and figure of merit calculated for different values of exchange interaction, as indicated. This figure was determined for a relatively low value of detuning from the particle-hole symmetry point, $\varepsilon = -0.48 U$, such that the exchange field effects rather compete than surpass other energy scales, and the system’s behavior is most interesting. First of all, one can note that below a certain temperature the conductance stops changing any more and retains its low-temperature value. This characteristic energy scale is set by the exchange field $\Delta \varepsilon _{\mathrm{exch}}$—when this field is larger than thermal energy, it determines the transport behavior and no further dependence on lowering *T* is visible, see Fig. 6. The Seebeck effect becomes then suppressed and so does the figure of merit. This can be understood by referring to the Sommerfeld expansion: once the exchange field is the dominant energy scale the conductance becomes constant and so does the low-energy transmission coefficient. Consequently, $[\partial \mathscr {T}(\omega ) / \partial \omega ]_{\omega =0} \approx 0$ and thus $S\approx 0$. This explains why $S\approx 0$ and $ZT\approx 0$ for $T/\Gamma \lesssim \Delta \varepsilon _{\mathrm{exch}}/\Gamma \approx 10^{-4}$, see Fig. 6b,d.

Let us now focus on the most interesting behavior, which is present for temperatures of the order and larger than the exchange field, and let us start with the case of ferromagnetic exchange interaction $J>0$. One can see that increasing *J* results in suppression of the low-temperature conductance, see Fig. 6a. This results from the fact that increasing *J* leads to lowering of $T_K$, and once $\Delta \varepsilon _{\mathrm{exch}}\gtrsim T_K$, the Kondo peak becomes suppressed. This is also visible in the thermopower and the figure of merit where a new maximum emerges at energy scale corresponding to the exchange field $T \approx \Delta \varepsilon _{\mathrm{exch}}$, see Fig. 6. While in the case of ferromagnetic exchange interaction mainly qualitative effects are visible, the case of antiferromagnetic exchange is completely different, see the dashed lines in Fig. 6. In this case, increasing |*J*| gives rise to the suppression of the conductance due to the formation of an antiferromagnetic spin state between the molecule’s orbital level and its magnetic core. When |*J*| is relatively low, the exchange field wins over the antiferromagnetic interaction and only a small suppression of *G* as a function of *T* is present, see e.g. the curve for $J=-0.3 T_K$ in Fig. 6a. However, further enhancement of |*J*|, stabilizes the antiferromagnetic state of the molecule and a full suppression of *G* is obtained once $J\lesssim -2T_K$. This behavior gives rise to a new maximum visible both in *S* and *ZT*. Moreover, while the maximum associated with the conductance drop as *T* is lowered moves to higher energies with decreasing *J* ($J<0$), there is an extra maximum visible just at the energy scale corresponding to $T \approx \Delta \varepsilon _{\mathrm{exch}}$. As a consequence, the temperature dependence of the Seebeck coefficient displays an interesting triple-peak structure, see Fig. 6b.

To complete the picture, in Fig. 7 we present the temperature dependence of *G* and *S* calculated for different values of magnetic anisotropy. Similarly to the case of nonmagnetic leads, we display the data for antiferromagnetic exchange interaction, which shows the most interesting behavior. Let us first analyze the case of uniaxial anisotropy, which is presented in the right column of Fig. 7. One can see that finite anisotropy gives rise to an enhancement of the low-temperature conductance. This is a consequence of the fact that anisotropy breaks the symmetry of the antiferromagnetic state of the molecule responsible for the conductance suppression. This effect gives rise to a local maximum in the Seebeck coefficient that develops at the energy scale of the order of magnetic anisotropy, see Fig. 7d. The case when the molecule exhibits easy-plane type of anisotropy is shown in the left column of Fig. 7. Now, one can observe a very strong dependence of both *G* and *S* on the magnitude of magnetic anisotropy. First of all, the low-*T* conductance exhibits a nonmonotonic dependence on $D<0$. Once the easy-plane anisotropy is present in the system and $|D|\lesssim 0.1 T_K$, *G* becomes suppressed. However, this tendency becomes reversed when $D\lesssim -0.1 T_K$, such that one observes an enhancement of *G* at low temperatures, see Fig. 7a. This is associated with the formation of a doublet ground state in the molecule in the case of considerable easy-plane anisotropy. Now, however, one witnesses a subtle interplay between the antiferromagnetic exchange interaction, the easy-plane magnetic anisotropy, the exchange field that splits the doublet state of the molecule and the Kondo correlations. The antiferromagnetic *J* gives rise to the suppression of *G* at low temperatures, which is however slightly hindered by the exchange field. On the other hand, turning on *D* ($D<0$), results initially in a larger suppression of the conductance, nevertheless, increased values of |*D*| eventually make the doublet state the ground state of the molecule, enhancing thus *G* due to the Kondo effect. Consequently, for sufficiently large |*D*|, the conductance shows a pronounced Kondo resonance, see Fig. 7a. The behavior of the conductance is clearly revealed in the temperature dependence of the Seebeck coefficient, which is shown in Fig. 7b. One can see that for values of *D* such that the conductance starts increasing, the thermopower exhibits a considerable maximum, which actually develops for $T\approx \Delta \varepsilon _{\mathrm{exch}}$. With further increase of |*D*|, this maximum becomes however decreased and its position moves towards higher temperatures of the order of the Kondo temperature, see Fig. 7b. This large enhancement of the Seebeck coefficient is a result of interplay between the intrinsic properties of the molecule, such as its magnetic anisotropy, and the ferromagnetism of the leads. Unfortunately, this effect is not associated with a particularly large figure of merit since the corresponding electrical conductance is then relatively low, see Fig. 7a.

### Spin Seebeck effect

When the electrodes are ferromagnetic and are characterized by a long spin relaxation time, in addition to the charge current, a spin current can be generated in the system^35,36^. The spin current $I_S$ flows if there is a difference between chemical potentials for given spin direction, i.e. in the presence of a spin bias $\Delta \mu _\uparrow \ne \Delta \mu _\downarrow$. The development of spin bias is conditioned by the spin relaxation time in the contacts compared to the time of tunneling events. The case of fast spin relaxation was discussed in previous section, now, let us focus on the situation when the spin accumulation can build up in the leads, i.e. when the spin relaxation time is long. In such case, the spin Seebeck effect $S_S$ can develop in the system. Assuming open circuit conditions, i.e. the vanishing of the spin and charge currents, it can be found from, $S_S = (S_\uparrow - S_\downarrow )/2$, where $S_\sigma$ is the thermopower in the spin channel $\sigma$, which yields^35,36^

9 $$\begin{aligned} S_S = -\frac{1}{2|e| T}\left( \frac{L_{1\uparrow }}{L_{0\uparrow }}-\frac{L_{1\downarrow }}{L_{0\downarrow }}\right) . \end{aligned}$$

On the other hand, the Seebeck coefficient in the case of finite spin bias is given by^35,36^, $S = -\frac{1}{2|e| T}\left( L_{1\uparrow }/L_{0\uparrow } + L_{1\downarrow }/L_{0\downarrow }\right)$, whereas the heat conductance can be expressed as, $\kappa =\frac{1}{T} \sum _\sigma \left( L_{2\sigma } - L_{1\sigma }^2/L_{0\sigma } \right)$.

We would like to emphasize that in the case of ferromagnetic leads in the absence of spin accumulation, the Seebeck effect is due to a generated voltage difference that is not spin dependent. However, the tunneling processes themselves do depend on spin, therefore one then observes the spin-dependent Seebeck effect. On the other hand, when the spin accumulation is relevant in the leads, a spin bias becomes generated, which gives rise to the spin Seebeck effect. Such spin thermopower results from the spin splitting of the chemical potentials in the contacts and is associated with the corresponding spin current. Moreover, we would like to note that spin Seebeck effect due to electron transport is also referred to as the spin-dependent Seebeck effect, whereas the typical Seebeck effect in the case of magnetic contacts is referred to as spin-resolved Seebeck effect^29,32^. However, in our paper we adopt the notion introduced by Świrkowicz et al.^35^.

**Figure 8:**
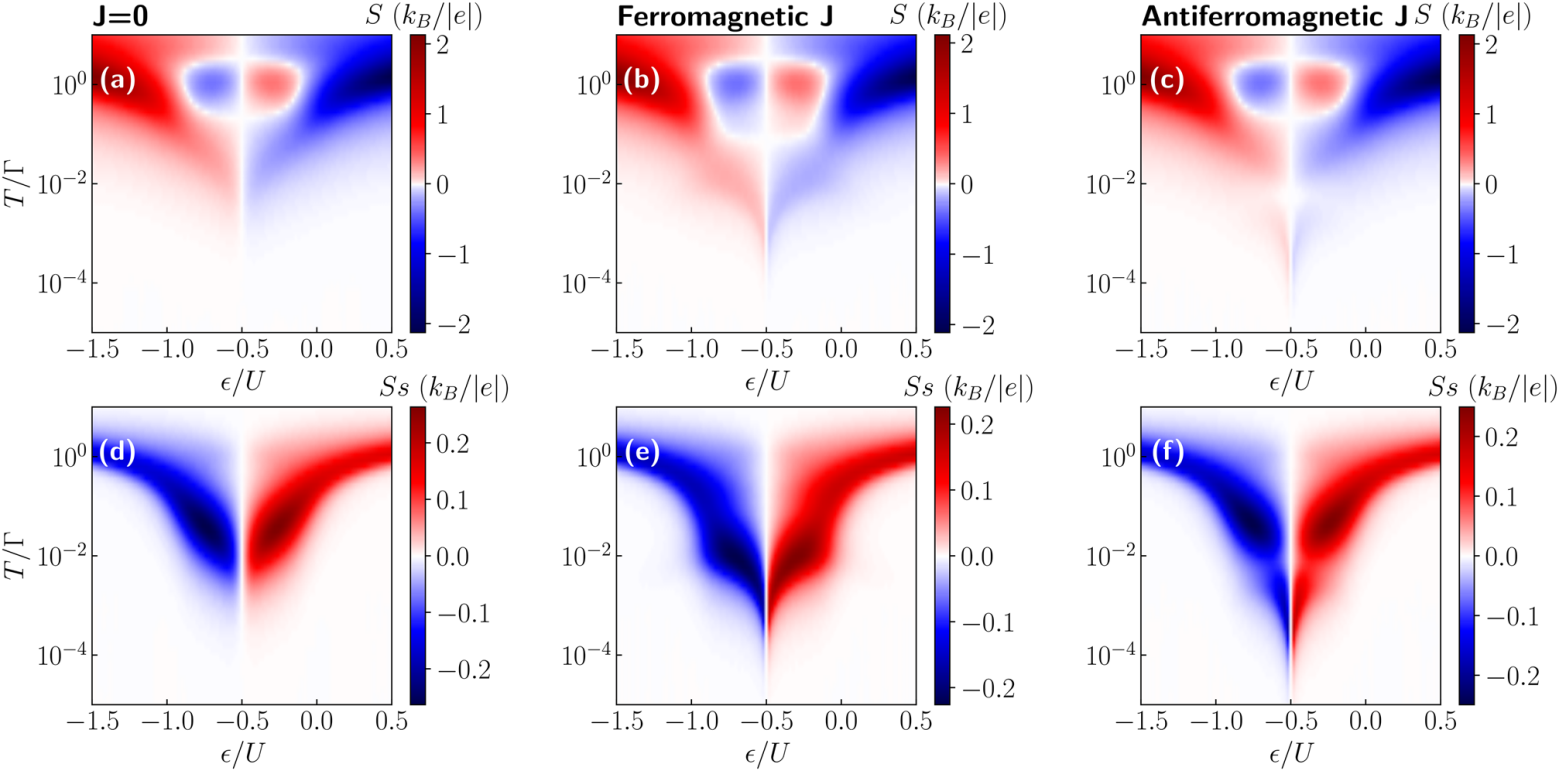
The spin Seebeck coefficient. (**a**–**c**) The Seebeck and (**d**–**f**) spin Seebeck coefficient as a function of temperature and orbital level position calculated for (**a**,**d**) $J=0$ (**b**,**e**) ferromagnetic *J* and (**c**,**f**) antiferromagnetic *J* in the case of finite spin accumulation in the leads. The other parameters are the same as in Fig. 5.

The Seebeck and spin Seebeck coefficients as a function of temperature and the position of the molecule’s orbital level calculated for different values of exchange interaction *J* are shown in Fig. 8. First of all, we note that the Seebeck effect at higher temperatures $T\approx \Gamma$ behaves generally very similarly as in the case of no spin accumulation in the leads, however, its low-temperature behavior is changed. While in the absence of spin accumulation, *S* exhibits an additional sign change for $-U\lesssim \varepsilon \lesssim 0$ and $T\lesssim 0.01 \Gamma$, in the case of long spin relaxation these features are not seen anymore. Instead, the Seebeck coefficient changes sign only once around $T\approx \Gamma$ and extends to low temperatures till it is quenched by the exchange field, see the first row of Fig. 8. On the other hand, the spin Seebeck effect displays completely different behavior. As can be seen, the only sign change occurs across the particle-hole symmetry point when tuning the position of the molecule’s orbital level. Moreover, the overall behavior is reversed as compared to the Seebeck coefficient. In the regions where *S* is generally negative $S_S$ is positive and vice versa. This is directly associated with the definition of *S* and $S_S$—while *S* captures the spin-resolved contributions due to hole and electron processes, $S_S$ presents mainly the difference between the spin-dependent components. As can be seen in the bottom row of Fig. 8, the spin-up contribution dominates the thermopower for $\varepsilon >-U/2$, while for $\varepsilon <-U/2$ the spin-down thermopower is dominant. Such behavior is visible in all considered cases, namely, for $J=0$ as well as for ferromagnetic and antiferromagnetic exchange interaction. However, there are some subtle differences. First of all, the spin Seebeck effect is finite for lower temperatures in the case of finite *J*, as compared to the case with $J=0$. Moreover, while for ferromagnetic *J*, $S_S$ exhibits a local maximum for $T\approx 0.01 \Gamma$, for antiferromagnetic *J*, there is a small local minimum in the temperature dependence of spin Seebeck effect, see Fig. 8f.

**Figure 9:**
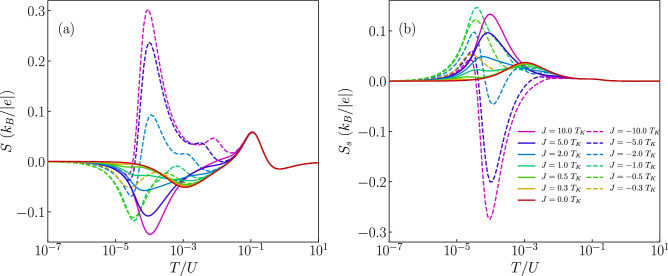
Spin thermopower for different values of exchange interaction. (**a**) The Seebeck and (**b**) spin Seebeck effect as a function of temperature for different values of exchange interaction *J*, as indicated. The parameters are the same as in Fig. 5 with $\varepsilon =-0.48U$.

The above-mentioned features are better visible in the temperature dependence of the thermopower and spin thermopower presented in Fig. 9. Moreover, it turns out that the above discussion is not fully complete, since one can now clearly observe that a sign change of both the Seebeck as well as spin Seebeck effect can develop, provided that the exchange interaction is sufficiently large. In the case of ferromagnetic exchange, a large minimum in *S* develops with increasing *J* at energy scale corresponding to the exchange field, see Fig. 9a. On the other hand, for the spin Seebeck effect a maximum forms at the same temperature for which *S* exhibits a minimum. An interesting behavior occurs for antiferromagnetic exchange interaction, when with increasing |*J*|, both the thermopower and spin thermopower change sign once $J\lesssim -T_K$. More specifically, *S* exhibits then a pronounced maximum, whereas a considerable minimum develops in $S_S$ for $T\approx \Delta \varepsilon _{\mathrm{exch}}$, see Fig. 9. The sign change of the thermopower is associated with the fact that the exchange field effects, which determine the sign of *S* and $S_S$ for low values of *J*, become overwhelmed by antiferromagnetic *J*, once the exchange interaction becomes sufficiently large, i.e. $|J|\gtrsim \Delta \varepsilon _{\mathrm{exch}}$.

**Figure 10:**
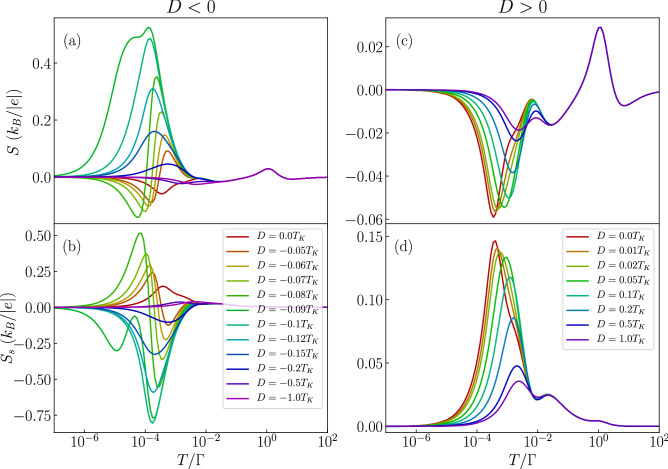
Spin thermopower for different values of magnetic anisotropy. The temperature dependence of (**a**,**c**) the thermopower and (**b**,**d**) the spin thermopower in the case of antiferromagnetic exchange interaction $J=-T_K$ for selected values of magnetic anisotropy *D*. The left (right) column presents the case of easy-plane (easy-axis) type of magnetic anisotropy. The parameters are the same as in Fig. 9.

To further understand the spin thermopower of magnetic molecules, we present the impact of magnetic anisotropy on the Seebeck and spin Seebeck effects for the case of antiferromagnetic exchange interaction in Fig. 10. First of all, for the case of uniaxial type of magnetic anisotropy, as shown in the right column of Fig. 10, there is a suppression in the minima (maxima) present in the Seebeck (spin Seebeck) effect with increasing anisotropy. This behavior is in fact similar to that observed in the case of no spin accumulation discussed in previous section. However, the case of an easy plane type of anisotropy presented in the left column of Fig. 10 shows far more interesting behavior. The thermopower and spin thermopower exhibit a change in sign around $T\approx \Delta \varepsilon _{\mathrm{exch}}\approx 10^{-4}$ for values of $D\lesssim -0.1 T_K$. For temperatures $T\lesssim \Delta \varepsilon _{\mathrm{exch}}$, a considerable peak, positive (negative) for the spin Seebeck (Seebeck) effect, is formed. On the other hand, for $T\gtrsim \Delta \varepsilon _{\mathrm{exch}}$ an additional maximum (minimum) develops for *S* ($S_S$), see the left column of Fig. 10. Further increase in the magnitude of anisotropy *D* suppresses the local extrema in the thermopower, as seen in the case of no spin accumulation. Consequently, one observes a strong nonmonotonic dependence of the (spin) Seebeck effect on the value of the easy-plane anisotropy. Large values of *S* and $S_S$ develop for such intrinsic parameters of the molecule that the temperature dependence of *G* is most spectacular, i.e. when the Kondo effect starts being restored with increasing |*D*|. However, once a plateau in *G* is formed at low temperatures due to the Kondo effect, which happens for considerable values of $D<0$, the thermopower becomes suppressed, see Fig. 10.

## Discussion

We have determined the thermoelectric properties of large-spin magnetic molecules attached to both nonmagnetic and ferromagnetic electrodes. Our analysis was focused on the strong correlation regime where the Kondo effect can emerge. To accurately address the system’s behavior in this nonperturbative regime, we have employed the numerical renormalization group method, which allowed us to study the electrical and heat conductances, the Seebeck effect and the figure of merit in the full parameter space of considered molecular junction. In particular, we have considered the cases of both ferromagnetic and antiferromagnetic exchange interaction *J* between the orbital level of the molecule and its magnetic core. Moreover, we have also analyzed the effect of finite magnetic anisotropy of the molecule.

In the case of nonmagnetic contacts, we have shown that the behavior of the Seebeck effect strongly depends on the type of exchange interaction *J*. In the case of ferromagnetic exchange, we have shown that the Seebeck coefficient displays features qualitatively similar to the case of $J=0$ (quantum dot case). However, due to a reduced Kondo temperature, the sign change in *S* occurs at a lower temperature and the thermopower was found to retain finite values extending to much lower *T* compared to the quantum dot case. Interestingly, in the case of antiferromagnetic exchange interaction, we have found a new sign change of the thermopower, at energy scale corresponding to the exchange interaction between the orbital level and molecule’s core spin. Moreover, we have also determined the influence of finite magnetic anisotropy on the thermoelectric properties of magnetic molecule. Finite magnetic anisotropy gives rise to new qualitative features especially in the case of antiferromagnetic *J*, where a new sign change of thermopower occurs in the case of easy-plane magnetic anisotropy.

On the other hand, when the leads are ferromagnetic, depending on the spin relaxation time, spin accumulation may be generated in the contacts giving rise to the spin Seebeck effect. First, we have focused on the impact of the spin-resolved tunneling on the thermopower in the absence of spin accumulation. We have shown that in the case of ferromagnetic exchange interaction there is an additional sign change in the temperature dependence of the thermopower. On the other hand, the low-temperature Seebeck effect has been found to strongly depend on the position of the molecule’s orbital level, which conditioned the strength of the exchange field in the molecule. Generally, the Seebeck effect becomes quenched once the temperature gets smaller than the corresponding exchange field.

Finally, we have assumed that the spin relaxation time in the leads is long, so that a spin bias can be generated in the system, resulting in the spin Seebeck effect. We have shown that for relatively low values of molecule’s exchange interaction the spin Seebeck effect changes sign only when tuning the orbital level across the particle-hole symmetry point. This is associated with the fact, that the sign of the spin thermopower is conditioned by the sign of the exchange field, which changes only when crossing $\varepsilon =-U/2$. However, if the molecule’s antiferromagnetic exchange interaction becomes larger than the Kondo temperature, the temperature dependence of the spin Seebeck effect can exhibit a sign change. This feature is associated with the interplay of exchange field, antiferromagnetic interaction between the orbital level and molecule’s core spin and the Kondo correlations. A similarly nontrivial behavior have been observed in the case of finite easy-plane magnetic anisotropy. We have found that the spin Seebeck effect exhibits then a nonmonotonic dependence on the magnitude of anisotropy. This effect is related to a revival of the Kondo effect when the anisotropy becomes large enough to bring about the two-fold degenerate ground state of the molecule, i.e. when the easy-plane anisotropy wins over both the exchange field as well as the molecule’s antiferromagnetic exchange interaction.

As far as the experimental progress is concerned, the field of molecular spin caloritronics is rather at its initial stage of development. The model studied here can however also describe quantum dots or impurities coupled to a large spin. It is therefore worth mentioning that, recently, there have been successful measurements of thermopower of Kondo-correlated quantum dots^10,25,26^. Moreover, spin-resolved electronic transport properties of molecular junctions have already been extensively studied^60,63–65^. Therefore, it seems that all the necessary ingredients are at hand and, with the state-of-the-art apparatus, it should be possible to explore the effects presented in this paper. We do hope that our work will foster further experimental efforts in this direction.

### Methods

The thermoelectric properties of the system in the linear response regime can be characterized by the Onsager integrals, $L_{n\sigma }$, which depend on the spin-resolved transmission coefficient $\mathscr {T}_{\sigma }(\omega )$. The transmission coefficient $\mathscr {T}_{\sigma }(\omega )$ can be related to the spin-dependent spectral function $A_{\sigma }(\omega )$ using the relation

10 $$\begin{aligned} \mathscr {T}_{\sigma }(\omega ) = \frac{4\Gamma _{L\sigma }\Gamma _{R\sigma }}{\Gamma _{L\sigma } + \Gamma _{R\sigma }}\pi A_{\sigma }(\omega ) , \end{aligned}$$

where $A_{\sigma }(\omega ) = -Im[G_{\sigma }^R(\omega )]/\pi$ and $G_{\sigma }^R(\omega )$ is the Fourier transform of the retarded Green’s function of the molecule’s orbital level, $G_{\sigma }^{R}(t)=-i\theta (t)\langle \{\hat{d}_\sigma (t),\hat{d}_\sigma ^\dagger (0)\}\rangle$. The main task is thus to accurately determine the spectral function of the system. One of the most powerful methods in this regard is the Wilson’s numerical renormalization group method^50,51,66,67^, which allows for nonperturbative treatment of all correlations in the system. In this method one performs a discretization of the conduction band with discretization parameter $\Lambda$ and, consecutively, a tridiagonalization of the Hamiltonian describing such discretized system is performed. Eventually, one obtains the following NRG Hamiltonian^50^

11 $$\begin{aligned} \hat{H}_{\mathrm{NRG}} = \hat{H}_{\text {mol}} + \sum _\sigma \sqrt{\frac{2W\Gamma _\sigma }{\pi }} \left( \hat{d}_{\sigma }^\dagger \hat{f}_{0\sigma } + \hat{f}_{0\sigma }^\dag \hat{d}_\sigma \right) + \sum _\sigma \sum _{n=0} \xi _n \left( \hat{f}_{n\sigma }^\dag \hat{f}_{n+1\sigma } + \hat{f}_{n+1\sigma }^\dag \hat{f}_{n\sigma }\right) , \end{aligned}$$

in which the molecule is coupled to the first site of the tight-binding chain with exponentially decaying hoppings $\xi _n$. Here, $\hat{f}_{n\sigma }^\dag$ creates a spin-$\sigma$ electron at the Wilson site *n* and *W* denotes the band halfwidth, which is used as energy unit $W\equiv 1$. This Hamiltonian is then solved in an iterative fashion by retaining a fixed number of states $N_K$. While the kept states are used to construct the statespace for the next iteration, the states that are discarded during calculation play a vital role in the problem as these states are used to construct the complete many-body basis of the full NRG Hamiltonian^68^. The discarded states are then used for the calculation of quantities of interest with the aid of the full density matrix^69^. In our calculations we have exploited the Abelian symmetries for the system’s spin *z*th component and charge. We have used $\Lambda = 2$ and kept at least $N_K=2000$ states in the iteration. Moreover, we have determined the Onsager transport coefficients from the raw NRG data without the need of broadening the Dirac delta peaks^36^.
